# Fasting and refeeding triggers specific changes in bile acid profiles and gut microbiota

**DOI:** 10.1111/1753-0407.13356

**Published:** 2023-01-22

**Authors:** Yi Zhang, Hongyan Qi, Long Wang, Chunyan Hu, Aibo Gao, Qihan Wu, Qiaoling Wang, Huibin Lin, Banru Chen, Xingyu Wang, Shuangyuan Wang, Hong Lin, Weiqing Wang, Yufang Bi, Jiqiu Wang, Jieli Lu, Ruixin Liu

**Affiliations:** ^1^ Department of Endocrine and Metabolic Diseases, Shanghai Institute of Endocrine and Metabolic Diseases Ruijin Hospital, Shanghai Jiao Tong University School of Medicine Shanghai China; ^2^ Shanghai National Clinical Research Center for Metabolic Diseases, Key Laboratory for Endocrine and Metabolic Diseases of the National Health Commission of the PR China, Shanghai Key Laboratory for Endocrine Tumor, State Key Laboratory of Medical Genomics Ruijin Hospital, Shanghai Jiao Tong University School of Medicine Shanghai China

**Keywords:** bile acid, biosynthesis, fasting, gut microbiota, reabsorption, refeeding, 胆汁酸, 肠道菌群, 禁食, 复食, 生物合成, 再吸收

## Abstract

**Background:**

Bile acids (BAs) are closely related to nutrient supply and modified by gut microbiota. Gut microbiota perturbations shape BA composition, which further affects host metabolism.

**Methods:**

We investigated BA profiles in plasma, feces, and liver of mice fed ad libitum, fasted for 24 h, fasted for 24 h and then refed for 24 h using ultraperformance liquid chromatography coupled to tandem mass spectrometry. Gut microbiota was measured by 16S rRNA gene sequencing. Expressions of BA biosynthesis‐related genes in the liver and BA reabsorption‐related genes in the ileum were analyzed.

**Findings:**

Compared with the controls, unconjugated primary BAs (PBAs) and unconjugated secondary BAs (SBAs) in plasma were decreased whereas conjugated SBAs in plasma, unconjugated PBAs, unconjugated SBAs and conjugated SBAs in feces, and unconjugated SBAs in liver were increased in the fasting mice. The expression of BA biosynthesis‐related genes in the liver and BA reabsorption‐related genes in the ileum were decreased in the fasting mice compared with the controls. Compared with the controls, *Akkermansia*, *Parabacteroides*, *Muribaculum*, *Eubacterium_coprostanoligenes* and *Muribaculaceae* were increased in the fasting mice whereas *Lactobacillus* and *Bifidobacterium* were decreased. All these changes in BAs and gut microbiota were recovered under refeeding. *Akkermansia* was negatively correlated with plasma levels of unconjugated PBAs, unconjugated SBAs and glucose, whereas it was positively correlated with plasma conjugated SBAs, fecal unconjugated PBAs, and fecal unconjugated SBAs.

**Conclusions:**

We characterized the BA profiles, gut microbiota, and gene expression responsible for BA biosynthesis and intestinal reabsorption to explore their rapid changes in response to food availability. Our study highlighted the rapid effect of nutrient supply on BAs and gut microbiota.

## Graphical abstract

**Highlights**

- For the first time, our study contributed a systematic evaluation of bile acid (BA) profiles in plasma, feces and liver tissue and gut microbiota changes triggered by fasting‐refeeding cycle in mice.
- Our work clearly demonstrated that overnight fasting leads to significant, but reversible changes in BA profiles and gut microbiota composition.
- Our results demonstrated that gene expression responsible for BA biosynthesis and intestinal reabsorption were suppressed under fasting status, whereas refeeding ameliorated this effect.
- Our study emphasized the important and rapid effects of nutrient supply on BA profiles and gut microbiota.

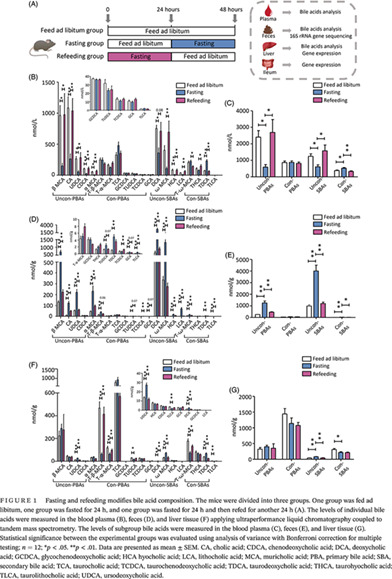

## 摘要

**背景:**胆汁酸(BAs)与营养供应密切相关, 并受到肠道菌群的影响。肠道菌群改变BAs的组成, 进而影响宿主的代谢。

**方法:**采用超高效液相色谱‐串联质谱(UPLC‐MS/MS)检测小鼠自由进食、禁食24 h、禁食24 h后再进食24 h的血浆、粪便和肝脏中的BAs谱。采用16S rRNA基因测序检测肠道菌群, 分析肝脏中BA生物合成相关基因和回肠中BA重吸收相关基因的表达情况。

**结果:**与对照组相比, 空腹小鼠血浆中未偶联的初级BAs (PBAs)和未偶联的次级BAs (SBAs)减少, 而血浆中偶联的SBAs、粪便中未偶联的PBAs和SBAs以及肝脏中未偶联的SBAs增加。与对照组相比, 空腹小鼠肝脏中BA生物合成相关基因和回肠中BA再吸收相关基因表达降低。与对照组相比, 空腹小鼠中阿克曼菌属、狄氏副拟杆菌、肠鼠杆菌、产粪甾醇真杆菌和拟杆菌增加, 乳杆菌和双歧杆菌减少。这些改变在复食条件下得以恢复。阿克曼菌属与血浆未偶联PBAs、未偶联SBAs和葡萄糖呈负相关, 与血浆偶联SBAs、粪便未偶联PBAs和粪便未偶联SBAs呈正相关。

**结论:**本研究通过分析BA谱、肠道菌群和与BA合成和肠道重吸收相关的基因表达, 探索其在食物供应中的快速变化。我们的研究强调了营养供应对BAs和肠道菌群的快速影响。

## 1 INTRODUCTION

Nutrient homeostasis, which is maintained by liver during fasting and feeding, is critical for health.^1^ Imbalance in nutrient homeostasis is a common trigger for the progression of metabolic diseases including obesity,^2^ type 2 diabetes mellitus (T2DM),^2^ nonalcoholic fatty liver disease,^3^ metabolic syndrome,^4^ and cardiovascular disease.^5^

Bile acids (BAs) are necessary for nutrient absorption, emulsification, and transport.^6^ In hepatocytes, BAs are derived from cholesterol catabolism, a process that involves the activation of two major pathways and at least 17 liver enzymes.^6^ The “classical pathway” is initiated by cholesterol 7α‐hydroxylase (CYP7A1), followed by the sterol 12α‐hydroxylase (CYP8B1) and enzymatic action of sterol 27‐hydroxylase (CYP27A1) to produce unconjugated primary BAs (PBAs), including chenodeoxycholic acid (CDCA) and cholic acid (CA). The “alternative pathway” is initiated by CYP27A1 and produces CDCA via oxysterol 7α‐hydroxylase (CYP7B1). In addition to CDCA and CA, mice also produce α muricholic acid (MCA), β MCA, and ursodeoxycholic acid (UDCA).^7^ In hepatocytes, most unconjugated PBAs are conjugated to either taurine (95% BAs are conjugated to taurine in mice) or glycine (predominant in human beings) by bile acid: CoA synthetase (BACS) and bile acid‐CoA: amino acid *N*‐acyltransferase (BAAT) and produce the conjugated PBAs prior to their secretion into intestine.^8^ In the gut, microbial enzymes from bacteria metabolize PBAs. Conjugated PBAs are deconjugated via bile salt hydrolases (BSH), 7α‐dehydroxylated, C‐6 epimerized and 6β‐epimerization to form unconjugated secondary BAs (SBAs), including lithocholic acid (LCA), deoxycholic acid (DCA), and ω MCA.^9^ Unconjugated SBAs are conjugated to taurine or glycine and then produce conjugated SBAs in liver.^7^ After food stimulation, BAs are released into the duodenum, and then both conjugated and unconjugated BAs are reabsorbed in the ileum by the apical sodium dependent BA transporter (ASBT)^10^ and organic solute transporter‐α/β (OST‐α/β).^8^ BAs are recirculated to the liver through the portal blood, a process known as enterohepatic circulation.^7^ Recently, BAs have also emerged as important signaling molecules in the control of energy^11^ and glucose metabolism.^12^ BAs regulate gut microbiome configuration,^7^ glycogen synthesis,^13^ hepatic gluconeogenesis,^14^ intestinal incretin secretion,^15^ and energy expenditure^16^ by binding to the nuclear hormone farnesoid X receptor^17^ and takeda G protein receptor 5^18^ in multiple organs. It has been found that in population‐based studies, impaired BAs metabolism is associated with the progression of metabolic disorder including T2DM,^19^ obesity,^20^ insulin resistance,^21^ and polycystic ovary syndrome.^22^ However, how nutrient availability systematically affects BA composition remain unclear. Maintenance of BA homeostasis based on nutrient supply may be a potential therapy to relieve metabolic disorders.

Growing evidence support the crosstalk between host health and gut microbiota. Alterations in gut microbiota composition are associated with various metabolic functions, including energy absorption, glucose homeostasis, and intestinal barrier homeostasis.^23^, ^24^, ^25^ Several studies have shown that the abundance of Firmicutes, Bacteroidetes, and butyrate‐producing bacteria such as *Akkermansia* is associated with metabolic disturbances, including T2DM and obesity.^23^, ^26^, ^27^, ^28^ Supplementation with *Akkermansia* improves gut‐barrier function and metabolic endotoxemia, thereby improving the systemic metabolism.^26^, ^29^, ^30^ Diet pattern (the type, amount, and timing of the meals) is a key factor that restructures the gut microbiota.^31^, ^32^, ^33^ Sustained caloric restriction (CR) during very‐low‐calorie diet (VLCD),^34^ intermittent fasting (IF),^35^ and long‐term fasting^36^ have an impact on the gut microbiome. Schwartzenberg et al. found weight loss caused by VLCD was associated with impaired nutrient absorption and *Clostridioides difficile* enrichment, and enrichment in *Clostridium difficile* was associated with decreased BAs levels.^34^ Shi et al. demonstrated BAs were potential mediators in the microbe‐host interaction involved in IF intervention.^35^ These studies emphasized the importance of diet‐microbiome‐BA interactions in regulating host metabolic homeostasis. However, the consequences of rapid changes in gut microbiota and BAs in response to nutrient supply for host pathophysiology have not been fully explored.

In this study, we contributed a systematic evaluation of changes of BA profiles (in plasma, feces, and liver), gene expression related to BA biosynthesis and intestinal BA reabsorption, as well as gut microbiota in mice fed ad libitum, fasted for 24 h, fasted for 24 h and then fed ad libitum for 24 h. We identified specific and rapid changes in BA components, microbial taxa, BA biosynthesis, and intestinal reabsorption‐related gene expression in response to nutrient availability. Of note, *Akkermansia* was increased in the fasting state and negatively correlated with plasma unconjugated PBAs, plasma unconjugated SBAs, and glucose levels, whereas it was positively correlated with plasma conjugated SBAs, fecal unconjugated PBAs, and fecal unconjugated SBAs. Hence, our research characterized the BA profiles, gut microbiota, gene expression related to BA biosynthesis, and intestinal reabsorption in fasting and refeeding mice, identifying the rapid changes in response to fasting‐feeding cycle. Our results thus highlighted the important and rapid effects of nutrient supply on BA profiles and gut microbiota.

## 2 MATERIALS AND METHODS

### 2.1 Animal care and experimental procedures

**FIGURE 1:**
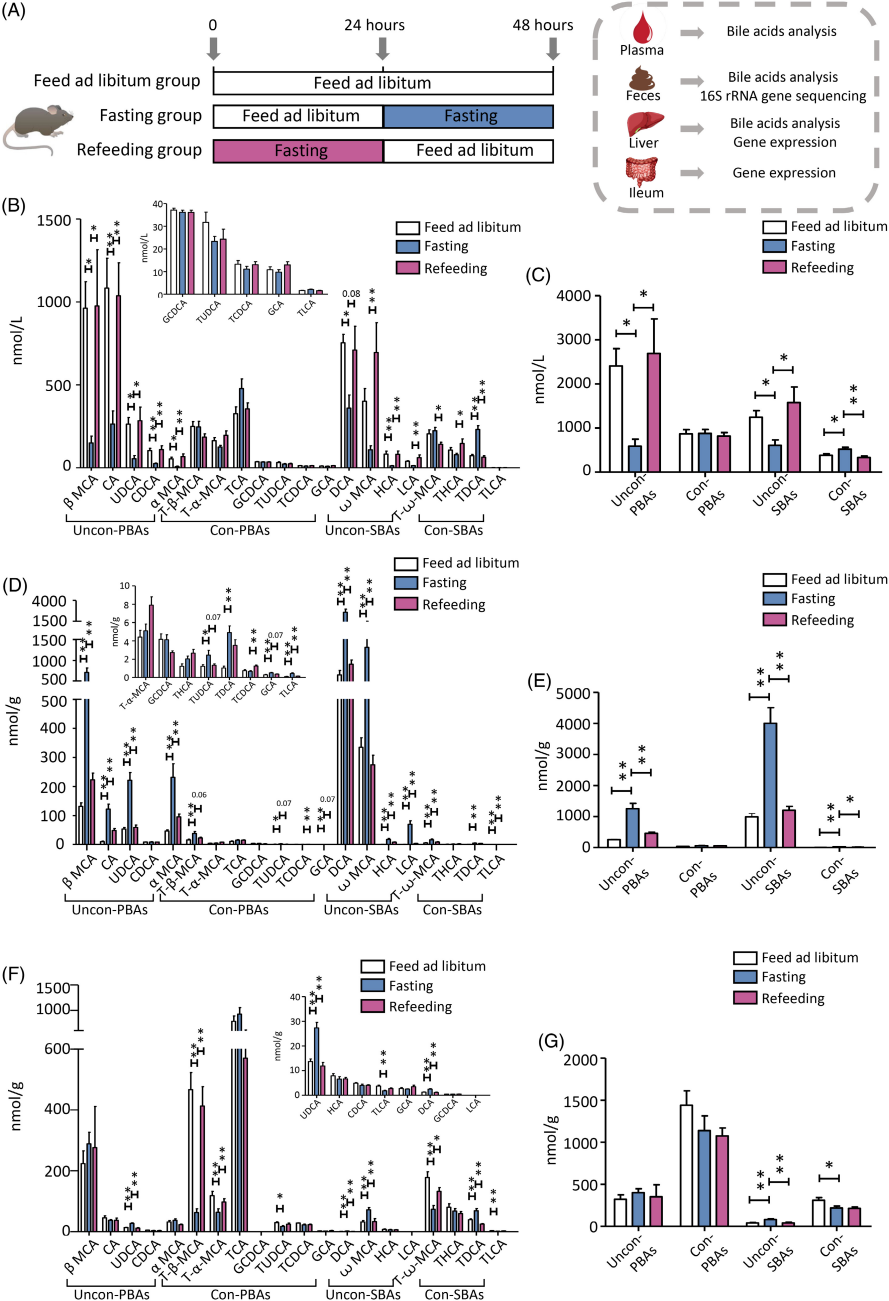
Fasting and refeeding modifies bile acid composition. The mice were divided into three groups. One group was fed ad libitum, one group was fasted for 24 h, and one group was fasted for 24 h and then refed for another 24 h (A). The levels of individual bile acids were measured in the blood plasma (B), feces (D), and liver tissue (F) applying ultraperformance liquid chromatography coupled to tandem mass spectrometry. The levels of subgroup bile acids were calculated in the plasma (C), feces (E), and liver tissue (G). Statistical significance between the experimental groups was evaluated using analysis of variance with Bonferroni correction for multiple testing; *n* = 12; *p < .05. **p < .01. Data are presented as mean ± SEM. CA, cholic acid; CDCA, chenodeoxycholic acid; Con‐, conjugated; DCA, deoxycholic acid; GCA, glycocholic acid; GCDCA, glycochenodeoxycholic acid; HCA, hyocholic acid; LCA, lithocholic acid; MCA, muricholic acid; T‐β‐MCA, tauro‐β‐muricholic acid; T‐α‐MCA, tauro‐α‐muricholic acid; T‐ω‐MCA, tauro‐ω‐muricholic acid; TCA, taurocholic acid; TCDCA, taurochenodeoxycholic acid; TDCA, taurodeoxycholic acid; THCA, taurohyocholic acid; TLCA, taurolithocholic acid; TUDCA, tauroursodeoxycholic acid; PBA, primary bile acid; SBA, secondary bile acid; UDCA, ursodeoxycholic acid; Uncon‐, unconjugated.

Specific‐pathogen‐free‐grade 6‐week‐old male C57BL/6 mice (*n* = 36) purchased from Shanghai Laboratory Animal Center were housed in groups of four mice per cage in a stable environment with free access to water. Mice were reared without any intervention for 1 week to acclimate to the new environment and then randomly divided into three groups: (a) mice in normal chow diet (NCD) fed ad libitum, (b) mice in NCD starved for 24 h, and (c) mice in NCD starved for 24 h and then fed ad libitum for 24 h (Figure 1A). The NCD was composed of 10% fat, 20% protein, and 70% carbohydrate. Mice were anesthetized with 10% chloral hydrate at the end of the experiment, and blood from the orbital plexus was collected in tubes. Blood samples were centrifuged at 3000 rpm, 4°C for 15 min. Plasma was separated and stored at −80°C for further biochemical testing. Using a blood glucose meter (LifeScan), blood glucose levels in mice were measured. The liver and ileum tissues of each mouse were dissected precisely and stored immediately at −80°C for subsequent analysis. Feces in the colon were collected precisely and stored immediately at −80°C for BA profiling and 16S rRNA gene sequencing. All these procedures were approved by the Animal Care Committee of Shanghai Jiao Tong University School of Medicine.

### 2.2 Bile acids analysis

BAs in plasma, feces, and liver tissue were measured. Detailed analytical methods are provided in the Supplementary Materials. BAs were quantified using ultraperformance liquid chromatography coupled to tandem mass spectrometry (UPLC‐MS/MS) in combination with multiple reactions monitoring methods on the ACQUITY UPLC system (Waters). All calibration standard solutions were mixed at appropriate concentrations and analyzed every 10 samples for quality control.

BAs were divided into four groups according to the degree of conjugation,^8^ including (a) unconjugated PBAs: UDCA, α MCA, β MCA, CA, and CDCA; (b) conjugated PBAs: tauroursodeoxycholic acid (TUDCA), tauro‐β‐muricholic acid (T‐β‐MCA), tauro‐α‐muricholic acid (T‐α‐MCA), glycochenodeoxycholic acid (GCDCA), taurochenodeoxycholic acid (TCDCA), glycocholic acid (GCA), and taurocholic acid (TCA); (c) unconjugated SBAs: DCA, LCA, hyocholic acid (HCA), and ω MCA; and (d) conjugated SBAs: taurolithocholic acid (TLCA), taurohyocholic acid (THCA), taurodeoxycholic acid (TDCA), and tauro‐ω‐muricholic acid (T‐ω‐MCA).

### 2.3 Sequencing the 16S rRNA gene and metataxonomic analysis

Using the E.Z.N.A.® Soil DNA Kit (Omega Bio‐tek), total microbial genomic DNA was extracted from feces. DNA quality and concentration were determined by 1.0% agarose gel electrophoresis and NanoDrop ND‐2000 spectrophotometer (Thermo Scientific Inc.). DNA was stored at −80°C for subsequent analysis. The V3‐V4 hypervariable region of the bacterial 16S rRNA gene was amplified by ABI GeneAmp® 9700 polymerase chain reaction (PCR) thermocycler (ABI) with primer pairs 338F (5'‐ACTCCTACGGGAGGCAGCAG‐3') and 806R (5’‐GGACTACHVGGGTWTCTAAT‐3').^37^ PCR reaction mix included 10 ng template DNA, 0.8 μl each primer (5 μM), 2 μl 2.5 mM dNTP, 0.2 μl BSA, 4 μl 5 × Fast Pfu buffer, 0.4 μl Fast Pfu polymerase, and ddH_2_O (20 μl final volume). All samples were amplified in triplicate. PCR amplification conditions were as follows: initial denaturation at 95°C for 3 min, amplification for 27 cycles (each cycle consists of denaturation at 95°C for 30 s, annealing at 55°C for 30 s and extension at 72°C for 45 s), single extension at 72°C for 10 min, and end at 4°C. PCR products were extracted from 2% agarose gel, then purified using the AxyPrep DNA Gel Extraction Kit (Axygen Biosciences) and quantified using Quantus™ Fluorometer (Promega). 16S rRNA gene sequencing data were processed using the Illumina MiSeq PE300/NovaSeq PE250 platform, details are provided in the Supplementary Materials.

### 2.4 RNA isolation and real‐time polymerase chain reaction

Total RNA was extracted from liver and ileum tissues using Eastep Super Total RNA Extraction Kit (Promega) and 1 μg of RNA was transcribed following the manufacturer's instruction by using the Reverse Transcription System Kit (Promega), which produced high‐quality complementary DNA. A real‐time PCR was performed on LightCyclerH 480 (Roche) using SYBR Green II Master (Takara). All mRNA quantification data were normalized to the housekeeping 36B4 gene. All samples were run in duplicate in a single 384‐well reaction plate, and data of the reaction were collected and analyzed according to the ∆∆CT method. Primers used in this study are listed in the Supplementary Materials.

### 2.5 Statistical analysis

Results are expressed as mean ± SEM. BA data were analyzed using one‐way analysis of variance (ANOVA) test followed by Bonferroni's post hoc test. Significant differences between the two groups were assessed by unpaired two‐tailed Student's *t* test. All of these data were tested for the normality before using Student's *t* test. For nonnormal distributions, nonparametric statistical analysis such as Mann–Whitney *U*‐test was used. GraphPad prism version 5.01 (GraphPad software) and SPSS Statistics version 23.0 (IBM SPSS) were used for statistical analyses. Using Spearman's correlation analysis, correlations between BA levels, glucose level, and abundance of gut microbiota were performed, and the post hoc correction was performed using the false discovery rate method. Differences between experimental groups were considered significant at *p* < .05. The Majorbio Cloud platform (https://cloud.majorbio.com) was used to carry out bioinformatic analysis of gut microbiota. See the Supplementary Materials for detailed analytical methods.

## 3 RESULTS

### 3.1 Fasting and refeeding modify bile acid composition

We first measured BA levels in plasma, feces, and liver tissues from the libitum fed mice, fasting mice, and refeeding mice (Figure 1A) using UPLC‐MS/MS. The baseline fecal BA levels (Figure S1) were comparable among the three groups. We found that the mice fasted for 24 h showed a significant decrease in plasma unconjugated PBAs (eg, α MCA, β MCA, UDCA, CA, and CDCA) and unconjugated SBAs (eg, DCA and HCA, despite ꙍ MCA and LCA showing a decreased trend) and a significant increase in conjugated SBAs (eg, TDCA), when compared with the libitum fed mice. It is noteworthy that these changes were completely recovered in mice refed for another 24 h. Nevertheless, plasma conjugated PBAs did not change in fasting‐refeeding cycle (Figure 1B,C).

Next, we found that fecal unconjugated PBAs (eg, α MCA, β MCA, UDCA, and CA), unconjugated SBAs (eg, ꙍ MCA, DCA, LCA, and HCA), and conjugated SBAs (T‐ꙍ‐MCA, TDCA, and TLCA) were significantly elevated in the fasting mice compared with the libitum fed mice. Although the level of conjugated PBAs subgroup did not change in fasting mice, T‐β‐MCA, TUDCA, and GCA were significantly increased. Similarly, these changes were overall recovered in mice after refeeding, back to the levels of those in the libitum fed mice (Figure 1D,E). Of note, unconjugated PBAs and unconjugated SBAs showed an opposite changing pattern whereas conjugated SBAs shared similar changing pattern in feces and plasma in response to the fasting‐refeeding cycle.

In liver, compared with the libitum fed mice, the levels of unconjugated PBAs subgroup and conjugated PBAs subgroup did not change in the fasting mice, whereas changes in individual BAs were observed. In the fasting mice, level of UDCA in unconjugated PBAs were significantly increased, whereas TUDCA, T‐α‐MCA, and T‐β‐MCA in conjugated PBAs were significantly decreased. The alterations of these BAs were reversed in the refeeding mice (Figure 1F,G). Unconjugated SBAs (eg, ꙍ MCA and DCA) were significantly increased, and conjugated SBAs (eg, T‐ꙍ‐MCA and TLCA) were significantly decreased in the fasting mice. These alterations were also recovered in mice after refeeding for 24 h (Figure 1F,G). Taken together, these results demonstrated a rapid and specific change in individual BA species and BA subgroups in plasma, feces, and liver in response to fasting‐refeeding status.

To determine which fecal BAs associated the most with plasma BAs under fasting‐refeeding condition, we further examined the correlation between BAs in plasma and feces (Figure S2). Levels of β MCA, CA, UDCA, α MCA, DCA, ꙍ MCA, HCA, and LCA in plasma were negatively correlated with their levels in feces, whereas levels of T‐ꙍ‐MCA, TDCA, and TLCA in plasma were positively correlated with their levels in feces (Figure S2).

### 3.2 The mRNA levels of genes related to bile acid biosynthesis and intestinal reabsorption under fasting‐refeeding cycle

**FIGURE 2:**
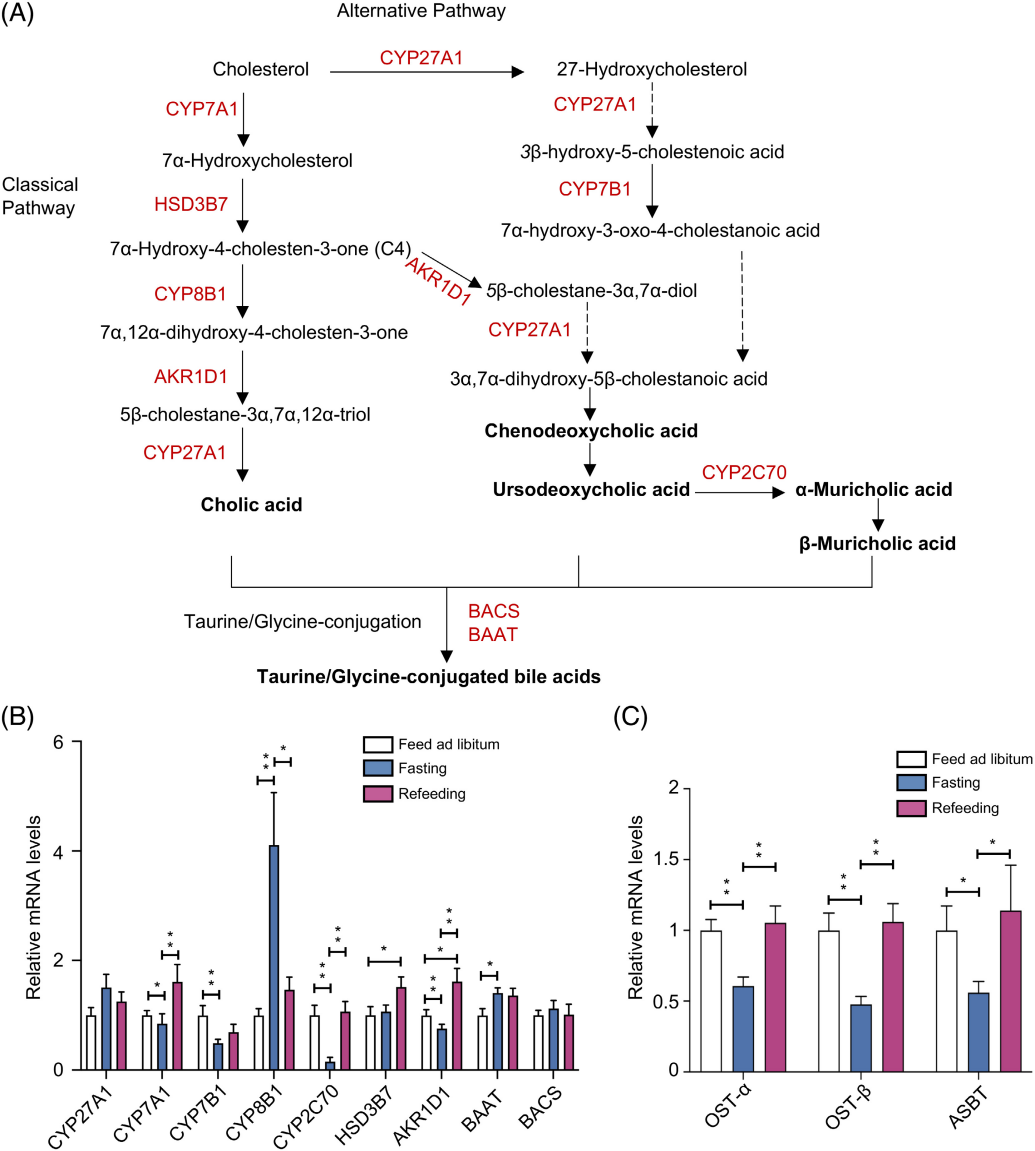
Bile acid biosynthesis and intestinal reabsorption under the fasting‐refeeding cycle. Bile acid (BA) synthesis pathways in liver (A). The enzymes required for the synthesis of cholic acid (CA) and chenodeoxycholic acid (CDCA). The classic pathway of  BA synthesis is initiated by the rate‐limiting enzyme cholesterol 7α‐hydroxylase (CYP7A1) to specifically hydroxylate cholesterol at the 7α position, forming 7α‐hydroxycholesterol. 7α‐hydroxycholesterol is converted to 7α‐hydroxy‐4‐cholesten‐3‐one (C4) by 3β‐hydroxy‐∆^5^‐C_27_‐steroid dehydrogenase (HSD3B7). Sterol 12α‐hydroxylase (CYP8B1) catalyzes 12α‐hydroxylation of C4 to 7α, 12α‐dihydroxy‐4‐cholesten‐3‐one, which is then converted to 5β‐cholestan‐3α, 7α, 12α‐triol by aldo‐keto reductase family 1 member D1 (AKR1D1). CYP8B1 is required for CA synthesis. Without CYP8B1, C4 is converted to 5β‐cholestan‐3α, 7α‐diol for CDCA synthesis. Mitochondrial sterol 27‐hydroxylase (CYP27A1) catalyzes the steroid side chain oxidation of 5β‐cholestan‐3α, 7α, 12α‐triol, which is subsequently converted to CA. The alternative BAs pathway in the liver is initiated by CYP27A1, which converts cholesterol to 27‐hydroxycholesterol and then to 3β‐hydroxy‐5‐cholestenoic acid. A nonspecific oxysterol 7α‐hydroxylase (CYP7B1) hydroxylates cholestenoic acid to 7α‐hydroxy‐3‐oxo‐4‐cholestanoic acid. In mouse liver, most CDCA is converted to α‐muricholic acid (α MCA) by a species‐specific sterol 6β‐hydroxylase (CYP2C70), and then the 7α‐OH group in α MCA is isomerized to a 7β‐OH group to form β MCA. MCAs are the primary BAs synthesized in mouse liver. The 7α‐OH group in CDCA can be isomerized to 7β‐OH to form ursodeoxycholic acid (UDCA). Primary unconjugated BAs are subsequently conjugated to glycine or taurine by BA: CoA synthetase (BACS) and BA‐CoA: amino acid *N*‐acyltransferase (BAAT) (A). Real‐time polymerase chain reaction (PCR) results of BA‐ biosynthesis‐related genes in liver tissue (CYP27A1, CYP7A1, CYP7B1, CYP8B1, CYP2C70, HSD3B1, AKR1D1, BAAT, and BACS) (B). Real‐time PCR results of intestinal reabsorption‐related genes in ileum tissue [organic solute transporter‐α (OST‐α), OST‐β and apical sodium dependent BA transporter (ASBT)] (C). A one‐way analysis of variance followed by Bonferroni's post hoc test was used to evaluate the significance of differences between the indicated groups; *n* = 12 (B and C). *p < .05, **p < .01.

Liver biosynthesis and intestinal reabsorption play a critical role in the regulation of enterohepatic circulation of BAs, BA pool size, and BA composition.^6^ We next analyzed the mRNA expression levels of BA biosynthesis‐related genes in liver and BA reabsorption‐related genes in ileum. Figure 2A summarized the BA biosynthesis pathways in liver. In hepatocytes, “classical pathway” involves CYP7A1, 3β‐hydroxy‐∆^5^‐C_27_‐steroid dehydrogenase (HSD3B7), CYP8B1, aldo‐keto reductase family 1 member D1 (AKR1D1) and CYP27A1, and “alternative pathway” involves CYP27A1 and CYP7B1. A species‐specific sterol 6β‐hydroxylase (CYP2C70) is required for the conversion of MCAs from CDCA in mouse liver. Unconjugated PBAs are subsequently conjugated to glycine or taurine by BAAT and BACS (Figure 2A).

In liver, the expression of CYP7A1, CYP7B1, CYP2C70, and AKR1D1 were significantly decreased in the fasting mice compared with the libitum fed mice, whereas the expression of CYP8B1 and BAAT were significantly increased, and all of these changes were completely recovered in the refeeding mice. The expression of CYP27A1 and BACS remained unchanged in the fasting‐refeeding cycle (Figure 2B). In ileum tissues, mice fasted for 24 h showed a significant decrease in the expression of OST‐α, OST‐β, and ASBT (intestinal reabsorption‐related genes) when compared with the libitum fed mice. The alterations in the expression of these genes were reversed in the refeeding mice (Figure 2C). These results together demonstrated that BAs biosynthesis in liver and intestinal reabsorption in ileum were suppressed under fasting status, whereas refeeding ameliorated this effect.

### 3.3 Gut microbiota features in fasting and refeeding

**FIGURE 3:**
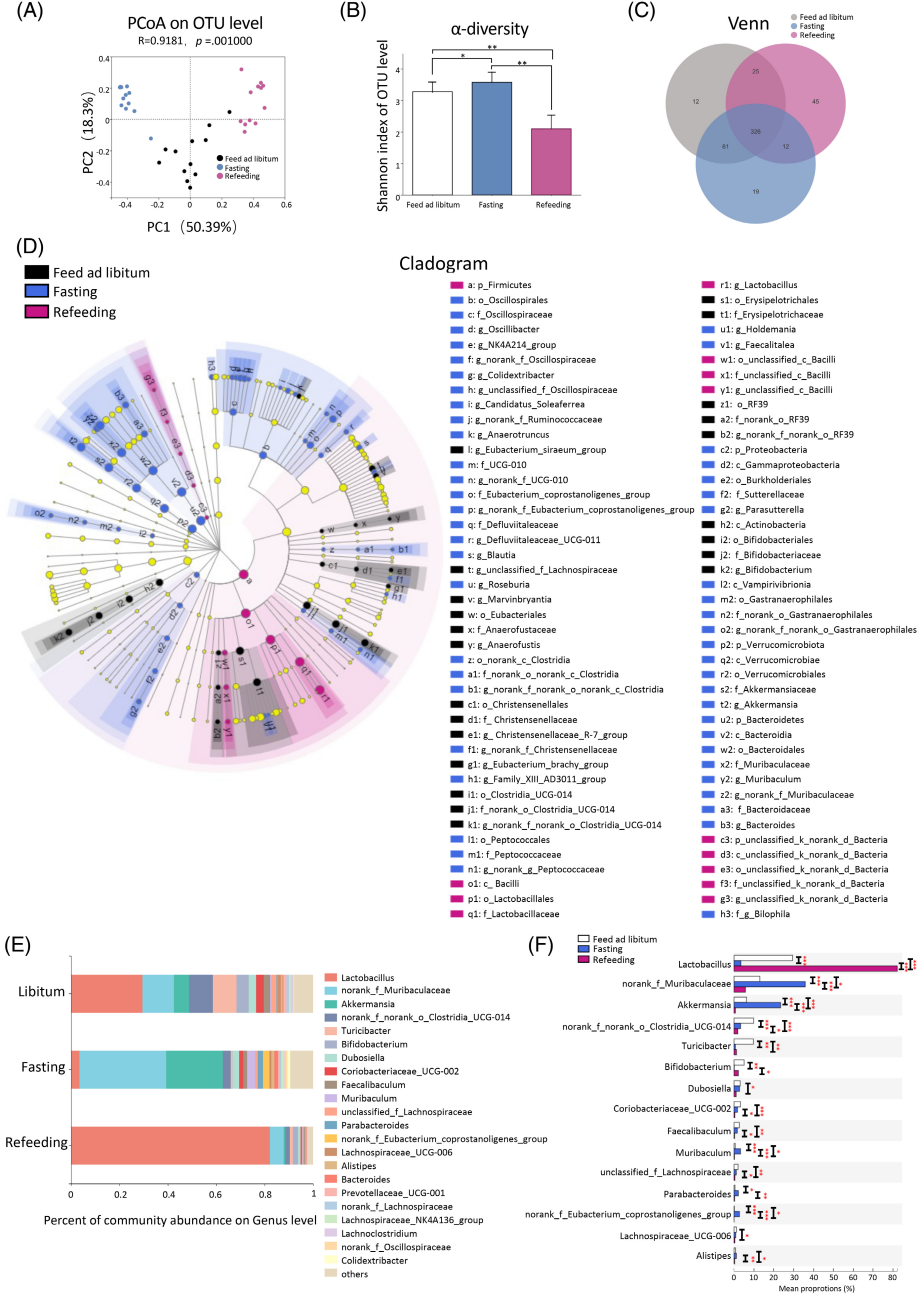
Gut microbiota features of fasting and refeeding. Principal coordinate analysis (PCoA) based on Bray–Curtis dissimilarity at the operational taxonomic units (OTU) level (A). α‐diversity (Shannon index) at the OTU level (B). Venn diagram for each OTU to compare the richness shared among three groups (C). The linear discriminant analysis effect size (LEfSe) results on the hierarchy induced by the taxa. Only significantly changed taxa names with the corresponding color are shown. The figure presents results with a linear determinant analysis (LDA) score more than 2 and a *p* value less than .05 (D). The relative abundance of gut microbiota at genus level. Genera that took up <1% of the microbiota were labeled together as “others” (E). Relative abundances of top 15 microbial genera that showed significant differences among three groups. A one‐way analysis of variance followed by Bonferroni's post hoc test was used to evaluate the significance of differences between the indicated groups (F). *p < .05, **p < .01, ***p < .001.

Using 16S rRNA gene sequencing, the characteristics of gut microbiota in libitum fed mice, fasting mice, and refeeding mice were analyzed. We first examined the baseline microbial features among the three groups and found that bacterial diversity (Shannon index, Figure S1) and the community structure (Figure S1) of gut microbiota were comparable among three groups at baseline. We further examined the gut microbiota of three groups with different nutrient status. Significantly, principal coordinate analysis (PCoA) indicated a distinct gut microbiota structure among the three groups (*p* = .001) (Figure 3A). Further, fasting mice had a higher bacterial diversity than libitum fed mice (Shannon index, *p* = .029, one‐way ANOVA test followed by Bonferroni's post hoc test), whreeas in refeeding mice, bacterial diversity was again decreased (Shannon index, *p* for refeeding mice and libitum fed mice = 1.011 × 10^−7^, *p* for refeeding mice & fasting mice = 2.98 × 10^−9^, one‐way ANOVA test followed by Bonferroni's post hoc test) (Figure 3B). Venn diagram showed that 326 operational taxonomic units (OTUs) were common to the three groups. 12 OTUs were unique for the libitum fed mice, 19 OTUs were unique for the fasting mice, whereas 45 OTUs were unique for the refeeding mice (Figure 3C). A list of the unique enriched bacterial genera among three groups is shown in the Supplementary Material (Figure S3). Overall, these results indicated that gut microbiota changed rapidly in response to food availability.

We used the linear discriminant analysis effect size (LEfSe) tool to identify specific communities within different groups (Figure 3D).^38^ In the libitum fed mice, six groups of bacteria were detected to be significantly enriched, namely Actinobacteria (the class, including its order Bifidobacteriales, its family Bifidobacteriaceae, and its genus *Bifidobacterium*), RF39 (the order, including its family and genus), Erysipelotrichales (the order, including its family Erysipelotrichaceae), Clostridia_UCG‐014 (the order, including its family and genus), Christensenellales (the order, including its family Christensenellaceae and its genus *Christensenellaceae_R‐7_group*), and Eubacteriales (the order, including its family and genus) (Figure 3D). In the fasting mice, eight groups of bacteria were significantly enriched, namely Bacteroidetes (the phylum, including its class Bacteroidia, its order Bacteroidales, its family Muribaculaceae and Bacteroidaceae, its genus *Bacteroides* and *Muribaculum*), Verrucomicrobiota (the phylum, including its class Verrucomicrobiae, its order Verrucomicrobiales, its family Akkermansiaceae, and its genus *Akkermansia*), Vampirivibrionia (the class, including its order, family, and genus), Proteobacteria (the phylum, including its class Gammaproteobacteria, its order Burkholderiales, its family Sutterellaceae, and its genus *Parasutterella*), Peptococcales (the order, including its family and genus), Clostridia (from order to genus), Defluviitaleaceae (the family, including its genus), and Oscillospirales (the order, including its family Oscillospiraceae, UCG‐010 and Eubacterium_coprostanoligenes_group, its genus *Oscillibacter*, *NK4A214_group*, *Colidextribacter*, *Candidatus_Soleaferrea* and *Anaerotruncus*) (Figure 3D). In the refeeding mice, Firmicutes (the phylum, including its class Bacilli, its order Lactobacillales, its family Lactobacillaceae, and its genus *Lactobacillus*) were significantly enriched (Figure 3D).

Figures 3E depicted the microbial composition of each group at genus level. Of note, libitum fed mice and refeeding mice were dominated by the *Lactobacillus*. Fasting for 24 h increased the relative abundance of *norank_f_Muribaculaceae* and *Akkermansia* and decreased the relative abundance of *Lactobacillus* and *Bifidobacterium*, compared with the libitum fed mice. These alterations were reversed in mice after refeeding for 24 h (Figure 3E). The top 15 abundant genera that differed among three groups were shown in Figure 3F. Of note, the relative abundances of seven dominant genera differed significantly between libitum fed mice and fasting mice. *Lactobacillus* (29.49% versus 3.44%, *p* < .001) and *Bifidobacterium* (5.04% versus 0.09%, *p* < .001) were significantly decreased, whereas *norank_f_Muribaculaceae* (13.00% versus 35.83%, *p* < .001), *Akkermansia* (6.25%versus 23.43%, *p* < 0.001), *Muribaculum* (0.59% versus 3.24%, *p* < .001), *Parabacteroides* (0.48% versus 2.14%, *p* < .05), and *norank_f_Eubacterium_coprostanoligenes_group* (0.11% versus 2.69%, *p* < .001) were significantly increased in fasting mice, compared with libitum fed mice. These alterations were also reversed in mice after refeeding for 24 h (Figure 3F). Taken together, these results illustrated that rapid change in nutrient supply profoundly affected the composition of gut microbiota in mice.

### 3.4 Associations of BAs and plasma glucose with gut microbiota in fasting and refeeding

**FIGURE 4:**
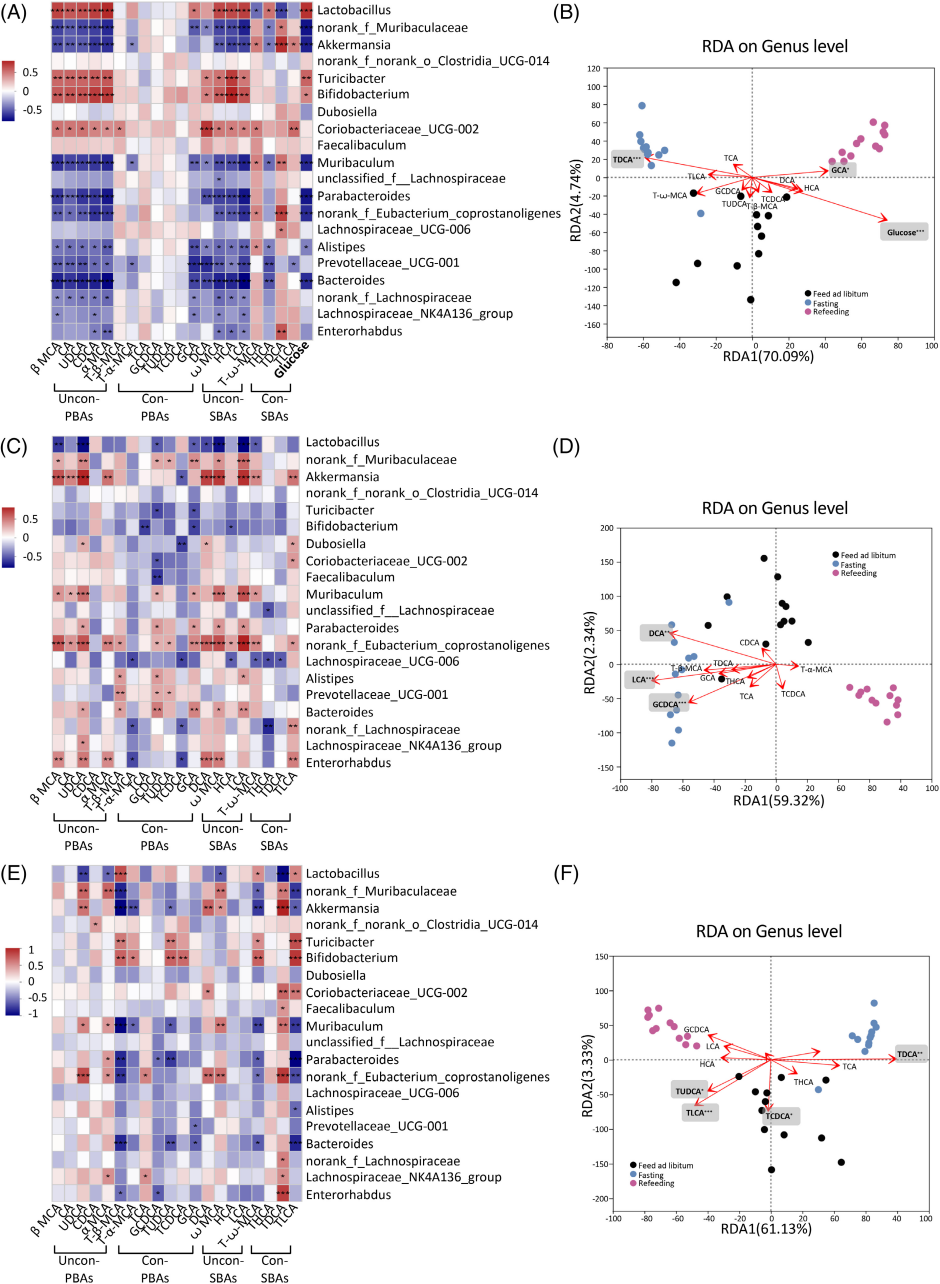
Associations of bile acid and glucose with gut microbiota. Spearman's correlations of the bile acids concentration in plasma (A), feces (C), and liver tissue (E) with the relative abundance of top 20 microbial genera. The gradient colors represent the correlation coefficients, with red color being positive and blue color indicating negative. *p < .05, **p < .01, ***p < .001 (Spearman's correlation after the post hoc correction using the false discovery rate method). Redundancy analysis/canonical correlation (RDA/CCA) analysis of 16S rRNA gene sequencing data (symbols), bile acids (arrows) and glucose (arrow) in plasma (B), feces (D), and liver tissues (F). The values of axes 1 and 2 are the percentages explained by the corresponding axis. *p < .05, **p < .01, ***p < .001. PBA, primary bile acid; SBA, secondary bile acid; Con‐, conjugated; Uncon‐, unconjugated.

These results demonstrated that the fasting‐refeeding cycle changed gut microbial community and BA profiles. The effect of nutrient supply on microbial communities may potentially affect BAs and glucose characteristics. Therefore, we further investigated whether gut microbial community, BAs, and glucose are correlated during the fasting‐refeeding cycle (Figure 4). Spearman's correlation analysis and redundancy analysis/canonical correlation analysis (RDA/CCA) revealed that the levels of plasma BAs (Figure 4A,B), fecal BAs (Figure 4C,D), and liver BAs (Figure 4E,F) were correlated with the top 20 abundant gut microbes at genus level. *Lactobacillus* and *Bifidobacterium* were positively correlated with plasma unconjugated PBAs (β MCA, α MCA, UDCA, CDCA, and CA) and unconjugated SBA levels (LCA, DCA, HCA, and ꙍ MCA), whereas *norank_f_Muribaculaceae* and *Akkermansia* were negatively correlated with unconjugated PBAs and unconjugated SBA levels. *Akkermansia* showed different correlation patterns with conjugated SBA levels. It was positively correlated with the levels of T‐ꙍ‐MCA, TDCA, and TLCA and negatively correlated with THCA levels (Figure 4A). More importantly, plasma glucose level was positively correlated with *Lactobacillus* and *Bifidobacterium*, and it was negatively correlated with *norank_f_Muribaculaceae* and *Akkermansia* (Figure 4A). RDA/CCA revealed that the microbial community structure was closely related to BAs and glucose. After removal of the redundant variables 11 plasma BAs and blood glucose were chosen for RDA/CCA. Plasma TDCA (*p* = .001), GCA (*p* = .043), and glucose (*p* = .001) were significantly associated with the gut microbiota structure (Figure 4B). In feces, unconjugated PBAs levels were negatively correlated with *Lactobacillus* and positively correlated with *norank_f_Muribaculaceae*, *Akkermansia* and *Bifidobacterium*. Unconjugated SBAs were negatively correlated with *Lactobacillus* and positively correlated with *norank_f_Muribaculaceae*, *Akkermansia* (Figure 4C). After removal of the redundant variables, 11 fecal BAs were chosen for RDA/CCA. Fecal GCDCA (*p* = .001), DCA (*p* = .002), and LCA (*p* = .001) were significantly associated with the gut microbiota structure (Figure 4D). In liver, α MCA and UDCA were negatively correlated with *Lactobacillus*, and they were positively correlated with *norank_f_Muribaculaceae*. The level of UDCA was positively correlated with *Akkermansia*. *Akkermansia* was negatively correlated with T‐β‐MCA, T‐α‐MCA, and TUDCA and positively correlated with UDCA, DCA and ꙍ MCA (Figure 4E). After removal of the redundant variables, 11 liver BAs were chosen for RDA/CCA. Liver TUDCA (*p* = .018), TCDCA (*p* = .018), TDCA (*p* = .002), and TLCA (*p* = .001) were significantly associated with the gut microbiota structure (Figure 4F). These results demonstrated the important and rapid effects of nutrient supply on the correlations between BA profile, glucose, and gut microbiota.

## 4 DISCUSSION

Previous studies support the crosstalk among gut microbiota, BAs, and nutrient supply in host health homeostasis, and changes of gut microbiota and BA profiles are involved in the progression of metabolic diseases such as T2DM and obesity.^6^, ^7^ Thus, elucidating the specific changes of gut microbiota and BA profile in response to nutrient supply will provide new targets for preventing and treating metabolic disorders. In the current study, we characterized BA profile, gut microbiota and BA biosynthesis, and intestinal BA reabsorption‐related gene expression in response to rapid change of nutrient supply.

To the best of our knowledge, for the first time, we contributed a systematic assessment of BA profiles (in plasma, feces, and liver), gut microbiota and BA biosynthesis and intestinal BA reabsorption‐related genes expression changes triggered by fasting‐refeeding cycle in mice. We found that in the fasting mice, plasma unconjugated PBAs and plasma unconjugated SBAs decreased whereas plasma conjugated SBAs, fecal unconjugated PBAs, fecal unconjugated SBAs, fecal conjugated SBAs, and liver unconjugated SBAs were increased, compared with the libitum fed mice. All these changes in BA composition were completely recovered after refeeding. BA biosynthesis in liver and intestinal reabsorption were suppressed under fasting status, whreeas refeeding ameliorated the decrease in biosynthesis and reabsorption of BAs due to fasting. Correspondingly, comparing with the gut microbiota in the libitum fed mice, *Akkermansia*, *Parabacteroides*, *Muribaculum*, *norank_f_Muribaculaceae*, and *norank_f_Eubacterium_coprostanoligenes_group* were largely increased in the fasting mice whereas *Lactobacillus* and *Bifidobacterium* were decreased. Also, these microbial changes were recovered under refeeding. More importantly, *Akkermansia* was negatively correlated with plasma unconjugated PBAs, plasma unconjugated SBAs, and glucose levels, whereas it was positively correlated with plasma conjugated SBAs, fecal unconjugated PBAs, and fecal unconjugated SBAs. Glucose contributed the most to the changes of gut bacterial community structure, followed by plasma GCA and TDCA. These results highlighted the important and rapid effects of nutrient supply on BA profile, gut microbiota, BA biosynthesis, and intestinal reabsorption.

In our results, compared with the ad libitum fed mice, the levels of plasma unconjugated PBAs and unconjugated SBAs were decreased in the fasting mice, whereas fecal unconjugated PBAs, unconjugated SBAs, and conjugated SBAs were increased in the fasting mice. These BAs showed an opposite changing pattern in feces and plasma in response to the fasting‐refeeding cycle. This might be because of the alterations in hepatic biosynthesis and intestinal reabsorption in the fasting mice, which play a critical role in the regulation of enterohepatic circulation of BAs, BA pool size, and BA composition.^1^ Consistently, we found that overnight fasting repressed, whereas refeeding induced the expression of CYP7A1 (the rate‐limiting enzyme in the classic pathway of BA synthesis, determining the rate of BA synthesis), CYP7B1 (involved in alternative pathway), AKR1D1 (involved in both classic pathway and alternative pathway), and CYP2C70 (CDCA to α MCA conversion) in liver. The reduction in BA biosynthesis gene expression in liver may contribute to the reduced plasma unconjugated PBAs and unconjugated SBAs after fasting. On the other hand, we detected a decreased expression of intestinal reabsorption‐related genes (OST‐α, OST‐β, and ASBT) in the ileum of fasting mice, which represents an attenuated enterohepatic circulation after fasting and may result in more BA accumulation in intestine and feces. The suppression of hepatic BA biosynthesis and intestinal BA reabsorption may explain the opposite changing pattern of BAs in plasma and feces. In summary, the alterations of BA profiles in response to rapid nutrient supply probably reflects a combination of decreased liver biosynthesis, attenuated enterohepatic circulation, reduced gallbladder emptying of PBAs owing to decreased nutrient consumption,^39^ and the changes in the abundance of BA‐metabolizing taxa (changes in gut microbiota related to 7α‐dehydroxylase and BSH^35^ such as *Lactobacillus* and *Bifidobacterium* as uncovered in our study).

CR intervention induces a long‐term reduction in nutrition supply. A clinical trial including 80 postmenopausal women who were overweight or obese showed 8‐week VLCD intervention increased HCA level and decreased GCDCA, GDCA, DCA TCA, and TDCA levels in feces, compared with a 4‐week weight maintenance.^34^ Mice with 25% CR (25% less than the average daily calorie intake) for 14 days induced changes of BA profiles characterized by decreased TCA and TDCA levels in feces and increased CDCA, TUDCA, and TCA levels in plasma, compared with the ad libitum fed mice.^40^ The aforementioned studies emphasized a relatively longer‐term nutrient restriction induced significant changes in BA profiles. Setchell et al. demonstrated that serum unconjugated BAs (including LCA, UDCA, DCA, CDCA, and CA) increased after breakfast but returned to fasting level in the absence of lunch in two healthy subjects.^41^ These results were consistent with our findings in fasting and refeeding mice. For the first time, we observed a considerable number of BA species' changes in plasma, feces, and liver in response to rapid fasting‐refeeding cycle. Compared with preceding observations in sustained CR intervention studies,^34^, ^40^ a more substantial amounts of changes were found in overnight fasting status. The differentially changed BA profiles between overnight fasting and long‐term CR could be explained by the following: (a) fasting is a complete food depletion whereas CR is part of food depletion; (b) after long‐term intervention, compensated regulations may be induced (including regulation of key enzymes); and (c) different gut microbiome and phenotypic changes may also affect BA profiles.

Consistent with the effects of sustained CR^42^, ^43^ and IF,^44^, ^45^, ^46^ our study showed overnight fasting significantly increased α‐diversity of gut microbiota. Our results demonstrated that 24‐h fasting stimulated shifts in the abundance of several bacteria in feces, and these alterations were reversed upon refeeding. Of note, *Lactobacillus* and *Bifidobacterium* decreased whereas *Akkermansia*, *Parabacteroides*, *Muribaculum*, *Eubacterium*, and *Muribaculaceae* increased in mice fasted for 24 h, and these alterations were also found to be reversed in mice refed for 24 h. *Lactobacillus* and *Bifidobacterium* were involved in BA metabolism including BA deconjugation via BSH and 7α‐dehydroxylation via 7α‐dehydroxylase.^8^ The decreased levels of plasma unconjugated SBAs and increased levels of plasma conjugated SBAs matched well with decreased abundances of *Lactobacillus* and *Bifidobacterium* in fasting mice presented in our study. Thus, the bacterial contribution to the observed BA phenotype in response to rapid fasting‐refeeding cycle is likely connected with the regulation of the conversion of conjugated BAs. More importantly, our study showed overnight fasting also induced a marked, but reversible change in the relative abundances of *Muribaculum*, *Parabacteroides*, *Eubacterium*, and *Muribaculaceae*, which have not been reported before. Bacteria in the family *Muribaculaceae* and genus *Muribaculum* produce propionate as the final product of fermentation^47^ and respond positively to acarbose in mice.^48^ *Parabacteroides* was found to alleviates obesity by producing succinate and SBAs in mice.^49^ Previous studies have shown that *Eubacterium*, a representative family of butyrate‐producing species, can alleviate intestinal inflammation, T2DM and obesity symptoms by producing short‐chain fatty acids.^50^, ^51^ However, detailed connections among fasting‐induced changes in host BAs, gut microbiota, and metabolism homeostasis remain to be explored in the future.

Of note, we found *Lactobacillus* and *Bifidobacterium* decreased whereas *Akkermansia* increased in mice fasted for 24 h, and these alterations were reversed in refeeding mice, reflecting the rapid changes of these taxa to nutrient supply. Previous studies showed the abundance of *Akkermansia* was increased whereas the abundance of *Bifidobacterium* was decreased in wild type C57BL/6 mice fed NCD after IF and CR intervention,^42^, ^44^, ^46^ which were consistent with our results. In terms of the effect of diets on the abundance of *Lactobacillus*, previous studies have shown inconsistent results, with some studies suggesting that CR and IF interventions led to an increase in the abundance of *Lactobacillus* ^42^, ^43^, ^44^, ^45^ and others showing that IF led to a decrease in the *Lactobacillus*.^45^ Liu et al. found that mice underwent IF (alternative‐day feed deprivation, 15 cycles total) had a significant increase in the abundance of *Lactobacillus* and *Akkermansia* compared with the control mice.^44^ Zhang et al. investigated the effects of three CR regimens (20% CR, 40% CR, and 60% CR) for 4 weeks on the gut microbiome composition in mice, and they found that CR dose‐dependently reduced the relative abundances of *Bifidobacterium* and remarkably increased the relative abundances of *Lactobacillus* and *Akkermansia* compared with the mice fed ad libitum.^42^ Pan et al. reported a unique *Lactobacillus*‐predominated microbial community was attained in mice after 2‐week 30% CR.^43^ Zhang et al. showed 30% CR led to a significant increase in the *Lactobacillus*, whereas 5:2 IF (2‐day fasting followed by a 5‐day ad libitum period) regimen led to a significant reduction in the *Lactobacillus*.^45^ Li et al. evaluated the effects of three IF regimens for 1 month, that is, 12‐, 16‐, and 20‐h fasting per day, respectively, on the gut microbiome, and they reported that 16‐h fasting regimen led to an increased level of *Akkermansia*, and these effects disappeared after the cessation of fasting.^46^

We noticed that changes of microbiota taxa especially *Lactobacillus*, *Bifidobacterium*, and *Akkermansia* were closely related to the changes of BAs and glucose during the fasting‐refeeding cycle. *Lactobacillus* and *Bifidobacterium* were proved to be related to BA metabolism in previous studies.^8^ *Akkermansia* has been proven to improve obesity, T2DM, hepatic steatosis, intestinal inflammation, and different cancers in mice.^52^ In our study, *Akkermansia* was negatively correlated with plasma unconjugated PBAs, plasma unconjugated SBAs, and glucose levels, whereas it was positively correlated with plasma conjugated SBAs, feces unconjugated PBAs, and feces unconjugated SBAs. We highlighted the potential impact of *Akkermansia* on regulating BA metabolism during the fasting‐refeeding cycle. Several small sample size clinical studies have also demonstrated the change of BAs is closely related to the change of gut microbiota under CR diet intervention. Schwartzenberg et al. found VLCD‐induced *Clostridium difficile* enrichment was associated with decreased BAs levels.^34^ A prospective cohort study including 10 obese postmenopausal women showed VLCD‐induced microbiota taxa change, especially *Bifidobacterium*, has great impact on the SBAs metabolism pathway.^53^ They also found positive correlations between *Akkermansia* and allolithocholic acid (an isomer of LCA).^53^

Our correlation analysis demonstrated that gut microbiota showed closer associations with plasma BAs than fecal BAs, suggesting changes in plasma BA profile were most closely related to the changes in gut microbiota during rapid fasting‐refeeding cycle. As BAs circulate, the majority (~ 95%) are actively absorbed across the distal small intestine wall.^6^ A small amount (~ 5%) escape ileal bile salt transport and enter the large intestine. In the large intestine primarily, but also in the small intestine, BAs are subjected to chemical modification by bacteria.^6^ Sayin et al. comprehensively mapped the BA profiles of C57BL/6 mice, and they found the serum BA profile most closely resembled the BAs in the distal small intestine rather than that in the colon and feces, which probably reflects the fact that most BAs are reabsorbed from the ileum.^54^ Thus, the BA profiles in feces were affected by many factors such as intestinal reabsorption rate and gut microbiota, which may explain the relatively weak association between fecal BA and gut microbiota. Future studies are needed to elucidate this phenomenon.

Furthermore, we found that the BA changes in plasma, feces, and liver during the fasting‐refeeding cycle may also contribute differently to microbial community. Plasam glucose contributed the most to the changes of gut microbiota, followed by plasma GCA and TDCA. LCA in feces and TDCA in liver contributed the most to gut microbiota remodeling. Thus, our study demonstrated that during rapid fasting‐refeeding cycle, there existed an interaction between BAs and gut microbiota, which could conceivably have a significant impact in the dietary intervention‐resulted metabolic outcome. A comprehensive assessment of BA profile‐gut microbiota crosstalk during the fasting‐refeeding cycle could provide insights into possible pathways that define the pathogenesis of metabolic diseases.

In summary, our work clearly demonstrated that overnight fasting led to significant, but reversible changes in BA profile in plasma, feces, and liver. Overnight fasting also induced reversible alterations in the gut microbiome structure. BA biosynthesis in liver and intestinal BA reabsorption were suppressed under fasting status, whereas refeeding ameliorated these changes. More broadly, this study emphasized the important and rapid impact of nutrient supply on BA profile and gut microbiota, allowing the mechanistic dissection of complex relationships between the host, microbiome, and BAs metabolism.

## DISCLOSURE

The authors declare that the study was conducted in the absence of any commercial or financial relationships that could be construed as a potential conflict of interest.

## Supporting information


**Data S1.** Supporting Information

**Figure S1.**Baseline data of bile acids profile and gut microbiota. Baseline data of fecal bile acids profile (A) among the three groups. Baseline α‐diversity (Shannon index) of the three groups at the operational taxonomic unit (OTU) level (B). Principal coordinate analysis (PCoA) based on Bray–Curtis dissimilarity at the OTU level (C). Statistical significance between the experimental groups was evaluated using analysis of variance with Bonferroni correction for multiple testing; *n* = 4; *p < .05. **p < .01. Data are presented as mean ± SEM.

**Figure S2.** Correlations between bile acids in plasma and feces. Spearman's correlations of the bile acids concentration in plasma and feces. The gradient colors represent the correlation coefficients, with red color being positive and blue color indicating negative. *p < .05, **p < .01 (Spearman's correlation after the post hoc correction using the false discovery rate method).

**Figure S3.** Venn diagram for the comparison of bacterial genera to identify common and uniquely enriched operational taxonomic units (OTUs) among the three groups.
