# Supplementary material for: Fasting and refeeding triggers specific changes in bile acid profiles and gut microbiota
Source: J Diabetes. 2023 Jan 22;15(2):165–80. doi: 10.1111/1753-0407.13356 (PMC9934961; doi:10.1111/1753-0407.13356)
Supplement: Supplementary file 1 — Data S1. Supporting Information [file JDB-15-165-s002.docx]

**Supplementary Material**

1. Supplementary materials and methods for bile acids measurement
2. Supplementary methods for 16S rRNA genes sequencing and metataxonomic analysis
3. ﻿Primers for quantitative real-time PCR
4. **Supplementary materials and methods for bile acids measurement**

*preprocessing of samples*

Plasma: Take 50 μL of plasma sample, add 200 μL of acetonitrile containing internal standard to extract the target compound and precipitate the protein, vortex, centrifuge (the internal standard and its concentration used for each compound are CA-d5 0.1 μg/mL, CDCA-d4 0.3 μg/mL, GCA-d5 0.2 μg/mL, GCDCA-d4 0.2 μg/mL, TCA-d5 0.1 μg/mL, TDCA-d5 0.1 μg/mL). 200 μL of the supernatant was lyophilized, reconstituted with 50 μL of 25 % acetonitrile aqueous solution.

Feces: Weigh about 10 mg of lyophilized fecal samples, grind them with grinding ball (25 Hz, 1 min), and add 1 mL of methanol extractant containing the internal standard (the internal standard and its concentration used for each compound are CA-d5 0.3 μg/ mL, CDCA-d4 0.9 μg/mL, GCA-d5 0.6 μg/mL, GCDCA-d4 0.6 μg/mL, TCA-d5 0.3 μg/mL, TDCA-d5 0.3 μg/mL, GCDCS-d5 0.6 μg/mL). Homogenize using a grinding ball (25 Hz, 1 min), centrifuge (14000 g, 4 ℃, 10 min), and take 800 uL of the supernatant for lyophilization. After lyophilization, the samples were reconstituted by 300 uL of 25% ACN/MilliQ, and filtered through a membrane filter.

Liver tissues: Liver tissue samples were lyophilized for 24 hours before weighing, and the dry weight of the tissue samples was weighed and recorded. Grinding balls and 400 μL of internal standard extractant were added (the internal standard and its concentration used for each compound are CA-d5 0.1 μg/mL, CDCA-d4 0.3 μg/mL, GCA-d5 0.2 μg/mL, GCDCA-d4 0.2 μg/mL, TCA-d5 0.1 μg/mL, TDCA-d5 0.1 μg/mL). Grind and extract, add 400 μL of chloroform, add 400 μL of water, vortex to mix, centrifuge, transfer the supernatant to a collection tube, and freeze-dry. After lyophilization, reconstitute with 50 μL of 25 % acetonitrile in water.

*Reagents*Sodium taurochenodeoxycholate (TCDCA), sodium glycocholate hydrate (GCA), sodium taurodeoxycholate (TDCA), chenodeoxycholic acid (CDCA), ursodeoxycholic acid (UDCA), taurocholic acid (TCA), sodium glycodeoxycholate (GDCA), glycoursodeoxycholic acid (GUDCA), cholic acid (CA), deoxycholic acid (DCA), sodium glycochenodeoxycholate (GCDCA), sodium tauroursodeoxycholate (TUDCA), lithocholic acid (LCA), cholic Acid-d4 (CA-d4) and NH4HCO3 were purchased from Sigma-Aldrich (St. Louis, MO, USA). Chenodeoxycholic acid-d4 (CDCA-d4), glycocholic acid-d5 (GCA-d5), lithocholylglycine (GLCA), tauro-α-muricholic acid (T-α-MCA), β-Muricholic Acid (β MCA) and tauro-β-Muricholic Acid-d4 (T-β-MCA-d4) were purchased from Toronto Research Chemicals Inc (North York, ON, Canada). Taurodeoxycholic acid-d5 (TDCA-d5) and taurocholic acid-d5 (TCA-d5) were purchased from International Laboratory (USA). Acetonitrile was purchased from Merk (Germany). Ethanol was purchased from Damao Chemical Reagent Factory (Tianjin, China).

*Ultraperformance liquid chromatography coupled to tandem mass spectrometry (UPLC-MS/MS) profiling*

Plasma, fecal and liver tissue samples collected were analyzed for bile acids (BAs) using the UPLC-MS/MS with multiple reactions monitoring methods in an ACQUITY UPLC system (Waters, USA) coupled to a triple quadrupole mass spectrometer (Waters, USA). For liquid chromatography separation, an UPLC BEH C8 column (100 mm× 2.1 mm i.d.; 1.7 μm; Waters, USA) was used at 40 °C and a flow rate of 0.2 mL/min. The mobile phases were 10 mM ammonium bicarbonate solution (A) and 100 % acetonitrile (B). The gradient elution was set at 25 % (v/v) B for 0.5 min, linearly increased to 40 % B during the next 12.5 min, linearly increased to 90 % B during the next 1 min, linearly decreased to 25% B during the next 0.5 min, and finally maintained at this composition for an additional 2.5 min. The injection volume was 5 µL. The full-scan MS spectra of selected precursor ions by multiple reaction monitoring (MRM) was obtained, and the product ions were used for identification and quantification of BAs. The MS/MS detection system was equipped with an electrospray ionization source (ESI) operating in negative ion mode. Briefly, nebulizing gas flow, 3 L/min, heating gas flow 10 L/min, interface temperature 300 ℃, DL temperature 250 ℃, heat block temperature 400 ℃, drying gas flow 10 L/min.

1. **Supplementary methods for 16S rRNA genes sequencing and metataxonomic analysis**

*Illumina MiSeq sequencing*

Purified amplicons were pooled in equimolar amounts and paired-end sequenced on an Illumina MiSeq PE300 platform/NovaSeq PE250 platform (Illumina, San Diego,USA) according to the standard protocols by Majorbio Bio-Pharm Technology Co. Ltd. (Shanghai, China).

*Data processing*

Raw FASTQ files were de-multiplexed using an in-house perl script, and then quality-filtered by fastp version 0.19.6^1^ and merged by FLASH version 1.2.7^2^ with the following criteria: (1) the 300 bp reads were truncated at any site receiving an average quality score of < 20 over a 50 bp sliding window, and the truncated reads shorter than 50 bp were discarded, reads containing ambiguous characters were also discarded; (2) only overlapping sequences longer than 10 bp were assembled according to their overlapped sequence. The maximum mismatch ratio of overlap region is 0.2. Reads that could not be assembled were discarded; (3) Samples were distinguished according to the barcode and primers, and the sequence direction was adjusted, exact barcode matching, 2 nucleotide mismatch in primer matching. Then the optimized sequences were clustered into operational taxonomic units (OTUs) using UPARSE 7.1^3^ with 97 % sequence similarity level. To minimize the effects of sequencing depth on α- and β-diversity measure, the number of 16S rRNA gene sequences from each sample were rarefied to 20,000, which still yielded an average Good’s coverage of 99.09 %, respectively.

The taxonomy of each OTU representative sequence was analyzed by RDP Classifier version 2.2^4^ against the 16S rRNA gene database (e.g. Silva v138) using confidence threshold of 0.7. The metagenomic function was predicted by PICRUSt2 (Phylogenetic Investigation of Communities by Reconstruction of Unobserved States)^5^ based on OTU representative sequences. PICRUSt2 is a software containing a series of tools as follows: HMMER was used to aligns OTU representative sequences with reference sequences. EPA-NG and Gappa were used to put OTU representative sequences into a reference tree. The castor was used to normalize the 16S gene copies.

*Statistical Analysis*

Bioinformatic analysis of the gut microbiota was carried out using the Majorbio Cloud platform (<https://cloud.majorbio.com>). Based on the OTUs information, rarefaction curves and α-diversity indices including observed OTUs, Shannon index and Good’s coverage were calculated with Mothur v1.30.1^6^. The similarity among the microbial communities in different samples was determined by principal coordinate analysis (PCoA) based on Bray-curtis dissimilarity using Vegan v2.5-3 package. The PERMANOVA test was used to assess the percentage of variation explained by the treatment along with its statistical significance using Vegan v2.5-3 package. The linear discriminant analysis (LDA) effect size (LEfSe)^7^ (http://huttenhower.sph.harvard.edu/LEfSe) was performed to identify the significantly abundant taxa (phylum to genera) of bacteria among the different groups (LDA score > 2, *P* < 0.05). Since there is a multicollinearity problem among the 20 bile acids species, the variance inflation factor (VIF) for each variable was estimated using the VIF function in the car package (<https://cran.r-project.org/web/packages/car/car.pdf>). The Redundancy analysis/canonical correspondence analysis (RDA/CCA) was performed using Vegan v2.5-3 package to investigate effect of bile acids and glucose on gut bacterial community structure. Forward selection was based on Monte Carlo permutation tests (permutations = 9999). Values of the x- and y-axes and the length of the corresponding arrows represented the importance of each bile acids species and plasma glucose in explaining the distribution of taxon across communities. Linear regression analysis was applied to determine the association between bile acids species and plasma glucose identified by RDA/CCA analysis and microbial α-diversity indices. The co-occurrence networks were constructed to explore the internal community relationships across the samples^8^. A correlation between two nodes was considered to be statistically robust if the Spearman’s correlation coefficient over 0.6 or less than -0.6, and the *P*-value less than 0.01. Heatmaps depicting spearman correlation or metabolic profile were generated using R studio (RStudio, Boston, MA, USA).

**References**

1. Chen S, Zhou Y, Chen Y, Gu J. fastp: an ultra-fast all-in-one FASTQ preprocessor. *Bioinformatics*. 2018; 34(17):i884-i890.DOI: 10.1093/bioinformatics/bty560.

2. Magoč T, Salzberg SL. FLASH: fast length adjustment of short reads to improve genome assemblies. *Bioinformatics*. 2011; 27(21):2957-2963.DOI: 10.1093/bioinformatics/btr507.

3. Edgar RC. UPARSE: highly accurate OTU sequences from microbial amplicon reads. *Nat Methods*. 2013; 10(10):996-998.DOI: 10.1038/nmeth.2604.

4. Wang Q, Garrity GM, Tiedje JM, Cole JR. Naive Bayesian classifier for rapid assignment of rRNA sequences into the new bacterial taxonomy. *Appl Environ Microbiol*. 2007; 73(16):5261-5267.

5. Douglas GM, Maffei VJ, Zaneveld JR, Yurgel SN, Brown JR, Taylor CM, et al. PICRUSt2 for prediction of metagenome functions. *Nat Biotechnol*. 2020; 38(6):685-688.DOI: 10.1038/s41587-020-0548-6.

6. Schloss PD, Westcott SL, Ryabin T, Hall JR, Hartmann M, Hollister EB, et al. Introducing mothur: open-source, platform-independent, community-supported software for describing and comparing microbial communities. *Appl Environ Microbiol*. 2009; 75(23):7537-7541.DOI: 10.1128/AEM.01541-09.

7. Segata N, Izard J, Waldron L, Gevers D, Miropolsky L, Garrett WS, et al. Metagenomic biomarker discovery and explanation. *Genome Biol*. 2011; 12(6):R60.DOI: 10.1186/gb-2011-12-6-r60.

8. Barberán A, Bates ST, Casamayor EO, Fierer N. Using network analysis to explore co-occurrence patterns in soil microbial communities. *ISME J*. 2012; 6(2):343-351.DOI: 10.1038/ismej.2011.119.

1. **Primers for quantitative real-time PCR**

| Genes | Primers |
| --- | --- |
| m-OSTα | *F* 5’-CCCTGACGGCATCTATGACC-3’ |
|  | *R* 5’-TGGCTTGACGGAAAAGGATGG-3’ |
| m-OSTβ | *F* 5’-GTATTTTCGTGCAGAAGATGCG-3’ |
|  | *R* 5’-TTTCTGTTTGCCAGGATGCTC-3’ |
| m-ASBT | *F* 5’-GTCTGTCCCCCAAATGCAACT-3’ |
|  | *R* 5’-CACCCCATAGAAAACATCACCA-3’ |
| m-CYP27A1 | *F* 5’-GCACAGGAGAGTACGGAGG-3’ |
|  | *R* 5’-CGGGCAAGTGCAGCACATA-3’ |
| m-CYP8B1 | *F* 5’-CACGGGGATGTCTTCACGG-3’ |
|  | *R* 5’-TGAGCACCAGTTCTTTTGCATAG-3’ |
| m-CYP7A1 | *F* 5’-AAACTCCCTGTCATACCACAAAG-3’ |
|  | *R* 5’-TTTCCATCACTTGGGTCTATGC-3’ |
| m-CYP7B1 | *F* 5’-AGCCGATTATCAGCGAAAGCC-3’ |
|  | *R* 5’-GCATCCAAAGGTTTGCCTTGT-3’ |
| m-CYP2C70 | *F* 5’-TCCCAAGGGCACAAGTGTAAT-3’ |
|  | *R* 5’-GCTGTAAGATGTTGGTCAGGATT-3’ |
| m-HSD3B7 | *F* 5’-AGGCCAGTCCAAAGACCATC-3’ |
|  | *R* 5’-TGCTCGTGTAGACCAGGTACT-3’ |
| m-BAAT | *F* 5’-GTGCTGGTGGATTGATGGAGT-3’ |
|  | *R* 5’-CCGAGGACCTTAGGATGTCTC-3’ |
| m-BACS | *F* 5’-TCTATGGCCTAAAGTTCAGGCG-3’ |
|  | *R* 5’-CTTGCCGCTCTAAAGCATCC-3’ |
| m-AKR1D1 | *F* 5’-AAGACAGCTATTGATGAGGGGT-3’ |
|  | *R* 5’-CCTCTTTACCTTCCCTTCTGCTA-3’ |
| m-36B4 | *F* 5’-GAAACTGCTGCCTCACATCCG-3’ |
|  | *R* 5’-GCTGGCACAGTGACCTCACACG-3’ |
