# Supplementary figures and images for: Fasting and refeeding triggers specific changes in bile acid profiles and gut microbiota
Source: J Diabetes. 2023 Jan 22;15(2):165–80. doi: 10.1111/1753-0407.13356 (PMC9934961; doi:10.1111/1753-0407.13356)

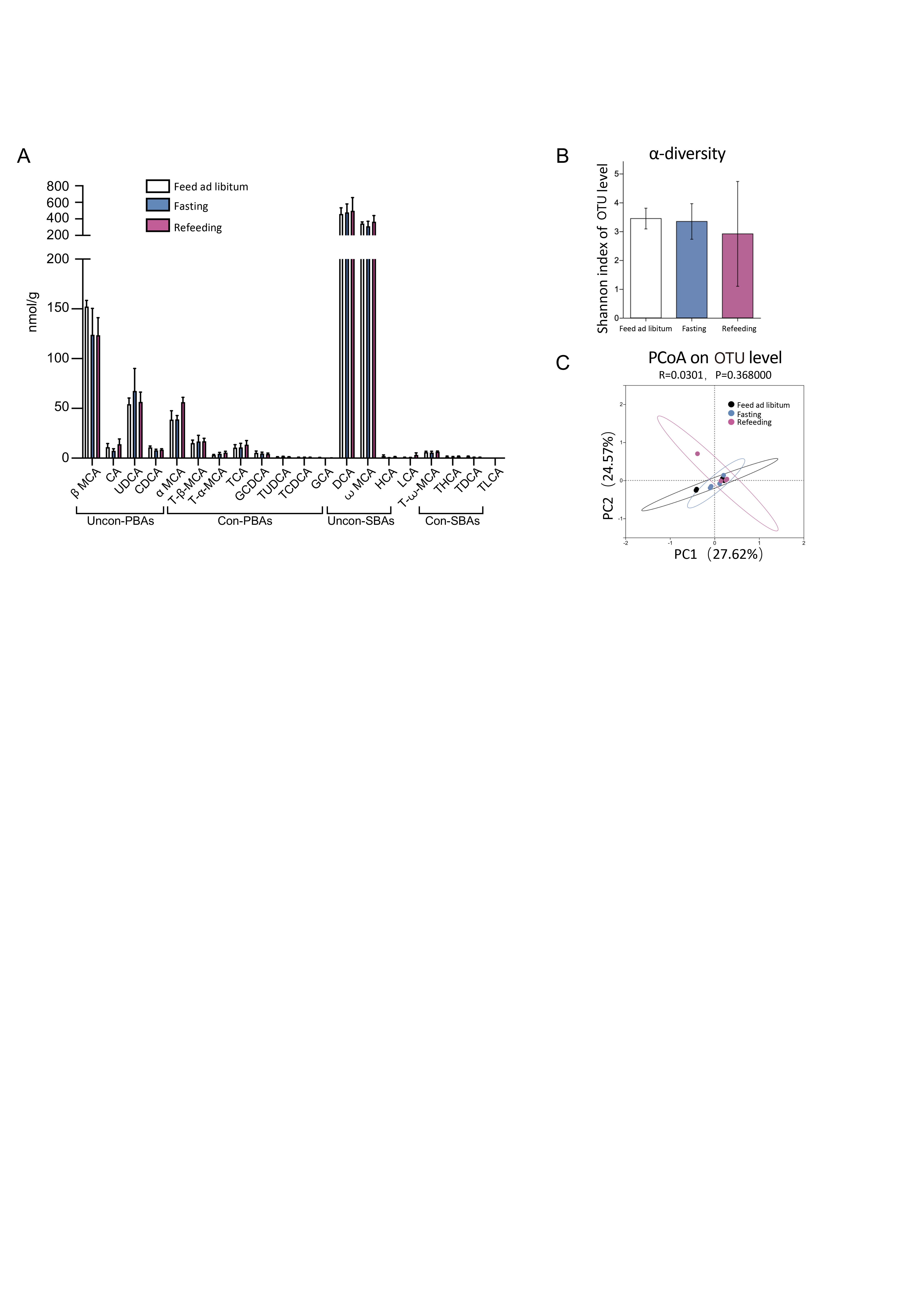

Supplement: Supplementary file 2 — Figure S1.Baseline data of bile acids profile and gut microbiota. Baseline data of fecal bile acids profile (A) among the three groups. Baseline α‐diversity (Shannon index) of the three groups at the operational taxonomic unit (OTU) level (B). Principal coordinate analysis (PCoA) based on Bray–Curtis dissimilarity at the OTU level (C). Statistical significance between the experimental groups was evaluated using analysis of variance with Bonferroni correction for multiple testing; n = 4; *p < .05. **p < .01. Data are presented as mean ± SEM. [file JDB-15-165-s001.tif]

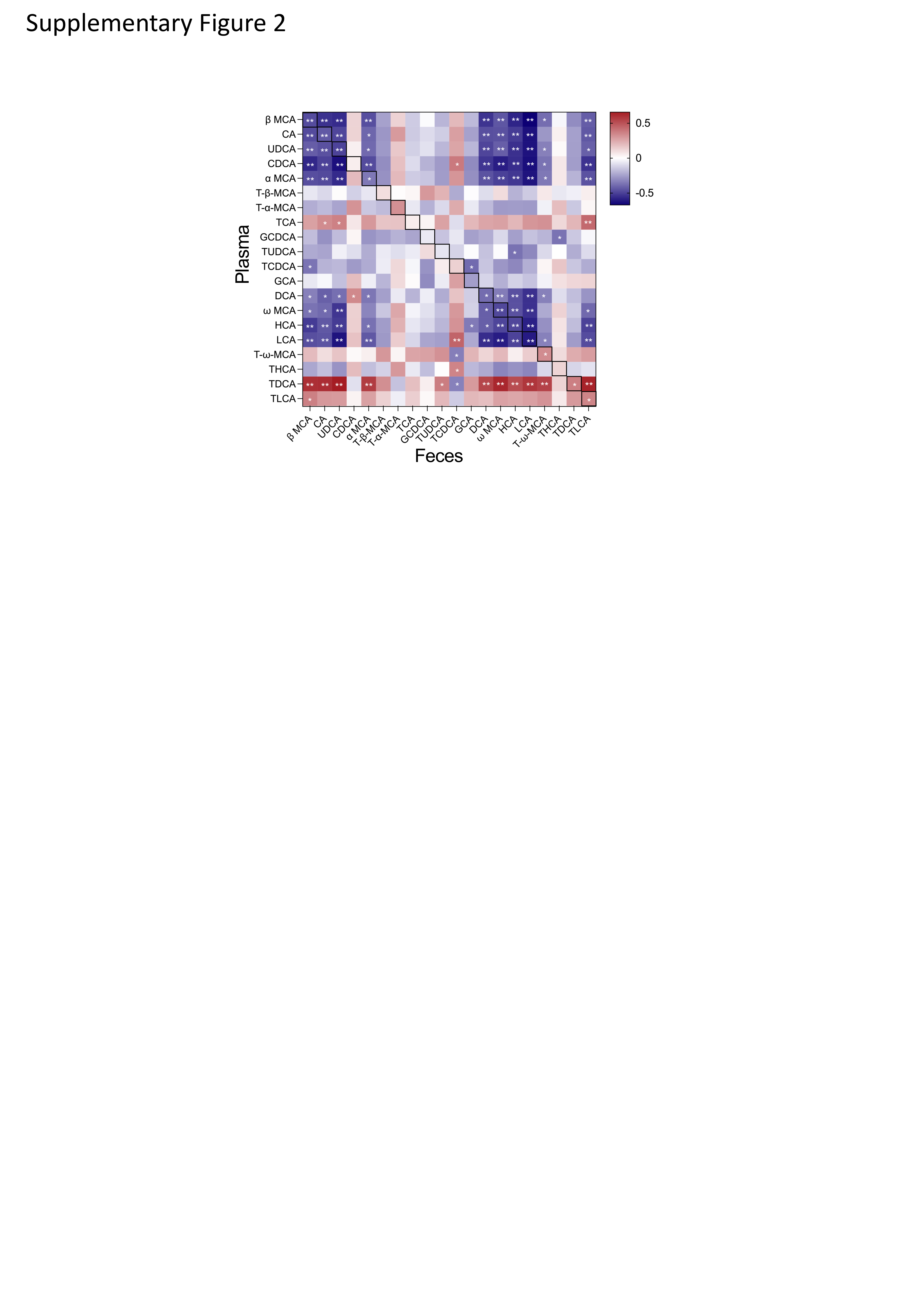

Supplement: Supplementary file 3 — Figure S2. Correlations between bile acids in plasma and feces. Spearman's correlations of the bile acids concentration in plasma and feces. The gradient colors represent the correlation coefficients, with red color being positive and blue color indicating negative. *p < .05, **p < .01 (Spearman's correlation after the post hoc correction using the false discovery rate method). [file JDB-15-165-s003.tif]

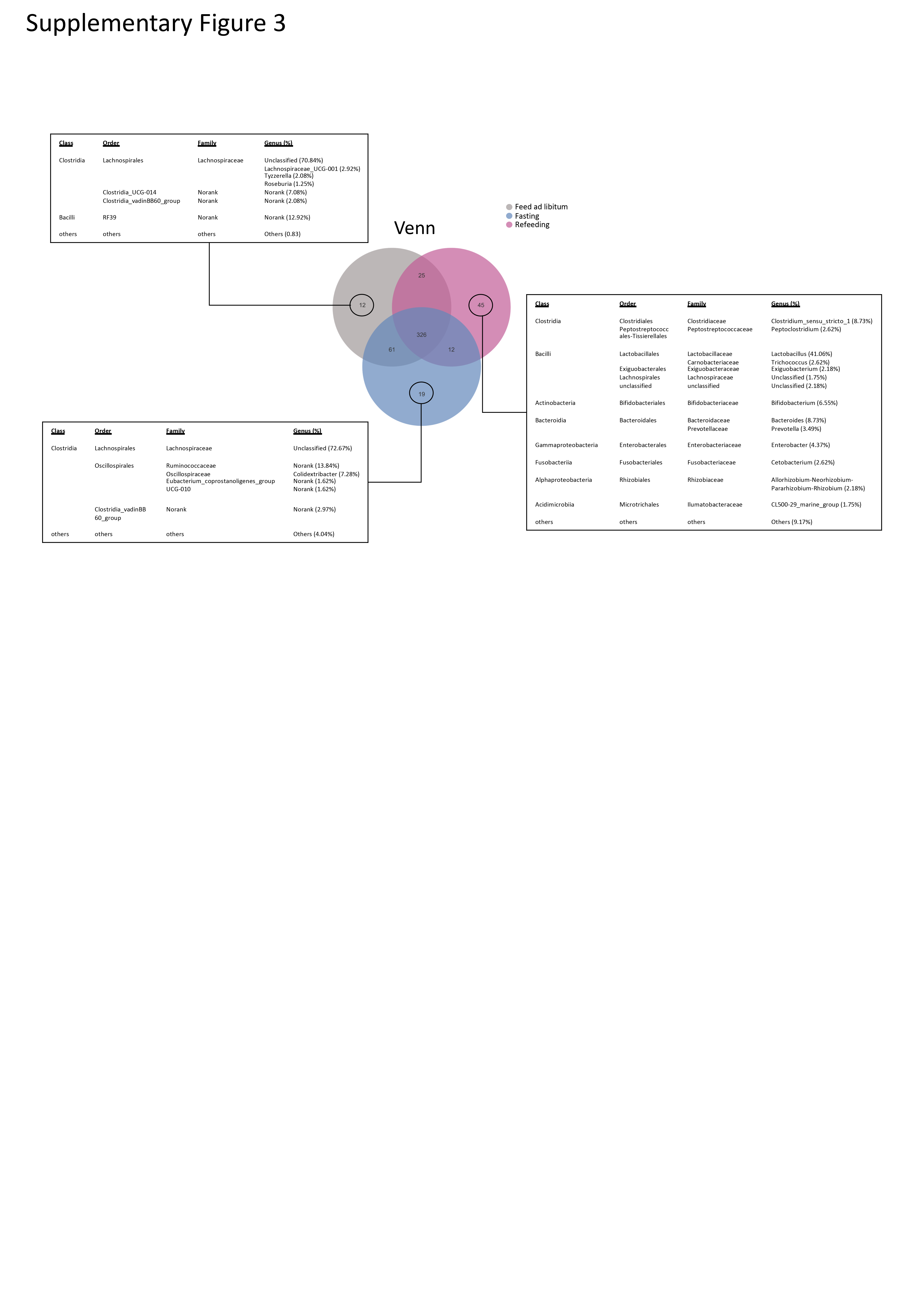

Supplement: Supplementary file 4 — Figure S3. Venn diagram for the comparison of bacterial genera to identify common and uniquely enriched operational taxonomic units (OTUs) among the three groups. [file JDB-15-165-s004.tif]
